# Developmental outcomes in 4-year-old children born in the first COVID-19 lockdown in England: preliminary findings from the Born In COVID Year – Core Lockdown Effects (BICYCLE) study

**DOI:** 10.1136/archdischild-2025-329529

**Published:** 2026-07-14

**Authors:** Nicola Botting, Chelo Del Rosario, Jane Flynn, Stian Reimers, Catherine Davies, Nayeli Gonzalez-Gomez, Alexandra Hendry, Elisabeth Hill, Michelle McGillion, Becky Moss, Laura Shapiro, Lucy A Henry

**Affiliations:** 1 School of Health and Medical Sciences, City St George’s University of London, London, England, UK; 2 School of Languages, Cultures and Societies, University of Leeds, Leeds, England, UK; 3 School of Psychology, Social Work and Public Health, Oxford Brookes University, Oxford, England, UK; 4 Department of Experimental Psychology, University of Oxford, Oxford, England, UK; 5 School of Psychology, University of Birmingham, Birmingham, England, UK; 6 Department of Psychology, University of Warwick, Coventry, England, UK; 7 School of Psychology, Aston University, Birmingham, England, UK

**Keywords:** Child Development, Covid-19, Psychology

## Abstract

**Background/objective:**

Children born during England’s COVID-19 lockdowns experienced markedly limited social exposure during a crucial developmental window. Given the importance of early interactions in shaping language, executive function and motor skills, this observational cross-sectional study examined developmental outcomes at age 4.

**Methods:**

205 children born in lockdown were assessed using standardised measures with published prepandemic norms: expressive vocabulary, expressive language and receptive language (Expressive Vocabulary, Word Structure and Sentence Structure subtests of the Clinical Evaluation of Language Fundamentals Preschool-second UK edition); receptive vocabulary (British Picture Vocabulary Scale, third edition); executive function (Global Executive Composite (GEC) and Emergent Metacognition Index (EMI) subscales of the Behaviour Rating Inventory of Executive Function-Preschool); motor skills (Motor Skills subscale from the Vineland Adaptive Behaviour Scales) and non-verbal reasoning (Matrices subtest from the British Ability Scales-3). Analyses (n=189–205) compared scores against prepandemic normative expectations and a within-sample benchmark of non-verbal reasoning to assess strengths and weaknesses against general cognitive development.

**Results:**

Compared with normative expectations, Sentence Structure (d=0.59), Expressive Vocabulary (d=0.24), receptive vocabulary (d=0.47) and Matrices (d=0.42) were significantly higher than expected for age. Caregiver-reported executive function was notably lower (GEC, d=–0.46; EMI, d=–0.36), while Word Structure (d=0.01) and Motor Skills (d=0.09) showed no differences. Benchmarked against non-verbal reasoning, Word Structure (d=−0.35), Expressive Vocabulary (d=−0.22), executive function (GEC, d=−0.72; EMI, d=−0.63) and Motor Skills (d=−0.33) were lower than cognitive potential. Sentence Structure (d=0.08) and receptive vocabulary (d=−0.01) were consistent with non-verbal reasoning.

**Conclusions:**

Caregiver-reported executive function was the most noteworthy area of concern for children born in lockdown. Expressive language and motor skills were lower than a benchmark of non-verbal reasoning, suggesting some impacts in relation to cognitive potential. This may lead to educational vulnerabilities and additional support needs in classrooms. Overall, these findings offer valuable early insight into postpandemic developmental impacts and underscore the urgent need for responsive educational policy and targeted support for those now in schooling.

**WHAT IS ALREADY KNOWN ON THIS TOPIC**

- We know that infants and young children born just before and during the COVID-19 pandemic had unusual and disrupted early lives, which may have impacted their early developmental outcomes.

**WHAT THIS STUDY ADDS**

- This study looks at key developmental outcomes of 4-year-old children in a tightly defined group that was born during the first strict lockdown in England.
- We assessed children via direct testing and caregiver reports using standardised measures of language, executive function and motor skills.
- We demonstrate that language and motor skills exceed or are as expected for age based on prepandemic normative data but that caregiver-reported executive function is a noteworthy area of concern.

**HOW THIS STUDY MIGHT AFFECT RESEARCH, PRACTICE OR POLICY**

- This study highlights the need for continued tracking of the cohort of children born during the COVID-19 lockdowns.
- Additional resources may be needed for school and family interventions to support the executive function needs of these children.

## Introduction

 COVID-19 was an unprecedented global event that profoundly affected children.^1^ Those born in lockdown in England faced a uniquely disrupted first year with severely restricted social interaction from 23 March 2020 to 19 July 2021 (https://instituteforgovernment.org.uk/sites/default/files/2022-12/timeline-coronavirus-lockdown-december-2021.pdf).^2 3^ Measures prohibited visiting of friends and family, mandated social distancing and face coverings and controlled time outside the home. Businesses, schools, baby groups and recreational spaces were closed. Family health professionals were routinely redeployed to COVID-related roles, and in-person appointments were rare.^3^

### Early development and social interaction

The first year of life is crucial for developing foundational language, executive function (EF) and motor skills,^4–8^ with some theoretical reviews suggesting a ‘functional dependency’ between social interaction and the development of EF.^9^ During this period, infants draw on multiple and varied interactions from a rich and diverse range of people and environments to optimally develop these skills.^10^ Babies born in lockdown experienced altered developmental environments for almost all of their first 18 months, spanning multiple strict lockdowns and intermittent restrictions, potentially impacting communicative experiences^11 12^ (different voices, unfamiliar individuals, seeing people’s faces and peer interaction), access to facilities supporting play and cognitive development (eg, early childcare settings) and opportunities for physical exploration. Family interactions in lockdown environments may also have changed in ways that ‘support or hinder language and socioemotional skill development’ (p108).^2^ Critically, language, EF and motor skills are intricately tied to school readiness and longer-term social and educational outcomes.^4–8^ Government developmental review data already suggests that the proportion of 2-year-olds achieving a ‘good level of development’ following COVID-19 in England fell from 84% in 2019 to 81% in 2022 and 79% in 2023.^13^

### Pandemic impacts on young children’s development

In existing literature on pandemic impacts, samples of included children have been born before the pandemic, during a wider pandemic timeframe or specifically during strict lockdown periods.

#### Children born before the pandemic

Communication outcomes for children aged 0–6 years during the pandemic show predominantly negative effects, although some studies report no impact or even benefits (see reviews^14 15^). Executive functioning in children aged up to 11 years when COVID-19 began was also affected,^16–22^ although findings are not uniform.^23^ Motor skills have been less frequently studied, but evidence broadly indicates impacts on fine and/or gross motor development in infants and young children.^24–26^

#### Children born during the wider pandemic timeframe

Larger-scale questionnaire-based evidence from the US broadly indicates pandemic impacts on early development between 6 and 24 months, including slightly lower communication (n=1024)^27^ and lower gross motor, fine motor and personal-social skills (n=217).^28^ Similar Canadian studies have found somewhat lower scores on communication, personal-social development and gross motor skills at 12 months (n=3742)^12^ and 24 months (n=4037)^29^ and lower EF (n=40).^30^ In an important smaller-scale study in Portugal (max n=37), where direct testing was employed, infants born between October 2020 and April 2021 were behind on language and communication at 12, 24 and 36 months.^31^ Other European studies have also reported increased risks of low language and declining developmental scores.^11 32^

#### Children born during strict lockdown periods

The CORAL study (Ireland) reported on a larger group of 917 children born in the initial lockdown (March–May 2020) using questionnaires. At 12 months, some delay in first words and social communication (eg, waving and pointing) was noted compared with a prepandemic cohort, although motor skills were not impacted.^33^ The same cohort showed delayed communication at 24 months, including following simple instructions, producing two-word phrases and using correct pronouns. Gross motor skills were lower in analyses unadjusted for key covariates, but other areas of development, including fine motor and problem-solving scales, were age-appropriate.^34^ A recent study from Bangladesh (n=526) also found children born in lockdown had lower motor skills at 20 months.^35^

### Present study

This paper presents interim findings from the Born In COVID Year – Core Lockdown Effects (BICYCLE) study on developmental outcomes in England in children aged 4 years: a key milestone as formal UK schooling begins. Children born in lockdown may demonstrate overall differences in development or may have strengths and difficulties in specific domains compared with general cognitive development. In language development research, especially, but also more widely for developmental conditions, identifying whether skills are in line with general ability often guides assessment and intervention. We report data from children born in lockdown to understand specific impacts on this cohort, to support their development and as a first step towards informing educational policy/practice in contexts of restricted early social opportunity.

This report:

1. Presents data on language, executive functioning, motor skills and non-verbal reasoning in 4-year-old children born during the strictest lockdown in England, in relation to expected levels for age (from published prepandemic normative data).
2. Investigates whether their language, executive functioning and motor skills are broadly in line with their general non-verbal reasoning ability to identify specific strengths and weaknesses against global development.

## Methods

The wider BICYCLE study is a cross-sectional observational study of the language and executive functioning abilities of 4-year-old children born before, during and after lockdown. The current study focuses on the subsample of children born in lockdown, 23 March 2020–23 June 2020, the initial COVID-19 lockdown in England.

### Recruitment and participants

Recruitment was via self-selected volunteer sampling but was stratified to represent the population across England’s geographical regions and postcode-based deciles of multiple deprivation (a proxy for socioeconomic status (SES)).^36^ Families were recruited through social media, school networks, community advertisements (shops and libraries) and word of mouth. Families were eligible if the parent/caregiver and child spoke English; the child was not born very preterm (<32 weeks), did not have known genetic disorders or global developmental delay and if the parent/caregiver was the primary caregiver from birth (as reported by caregivers during a two-stage consent process and through an introductory phone call). A total of 205 children took part (see figure 1 and table 1), although final assessment samples vary in number from 189 to 205 due to missing data on individual tasks.

**Figure 1:**
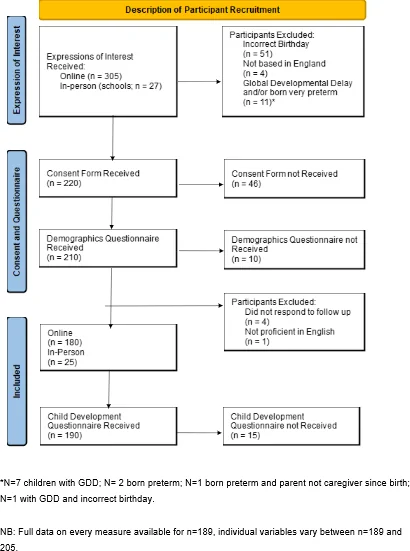
Recruitment process for the Born In COVID Year – Core Lockdown Effects study. GDD, global developmental delay.

**Table 1:** Demographic characteristics of the born-in-lockdown sample of 4-year-old children

Demographic variable	Mean (SD) (range)) or n (%)
Child age at assessment (months)	52.79 (3.38) (48.39–59.66)
Child gestational age (weeks)*	39.35 (1.84) (32–42)
Maternal age at time of child’s birth (years)†	32.61 (4.18) (19–43)
Child gender	
Male	120 (58.5%)
Female	85 (41.5%)
Child ethnicity	
White or White British	166 (81.0%)
Mixed or multiple ethnicity	19 (9.3%)
Asian or Asian British	12 (5.9%)
Black, Black British, Caribbean or African	6 (2.9%)
Other ethnicity	2 (1.0%)
Multiple languages (child)	
No	174 (84.9%)
Yes	31 (15.1%)
Location by region	
North West	25 (12.2%)
North East	6 (2.9%)
Yorkshire and Humberside	25 (12.2%)
East of England	21 (10.2%)
West Midlands	20 (9.8%)
East Midlands	6 (2.9%)
South West	24 (11.7%)
South East	28 (13.7%)
London††	50 (24.4%)
Index of Multiple Deprivation Decile	
1	11 (6.3%)
2	8 (4.5%)
3	17 (9.7%)
4	15 (8.5%)
5	14 (8.0%)
6	16 (9.1%)
7	20 (11.4%)
8	19 (10.8%)
9	18 (10.2%)
10	38 (21.6%)
Number of people in household	
2	3 (1.5%)
3	45 (22.0%)
4	119 (58.0%)
5	27 (13.2%)
6	7 (3.4%)
7 or more	4 (2%)
Number of siblings	
0	38 (18.5%)
1	125 (61.0%)
2	32 (15.6%)
3	6 (2.9%)
4 or more	4 (2.0%)
Number of people with parental responsibility	
1	8 (3.9%)
2 or more§	197 (95.6%)
Maternal education	
Up to GCSE or equivalent¶	10 (4.9%)
A-level or equivalent **	27 (13.2%)
Other vocational/qualification	7 (3.4%)
Undergraduate or postgraduate degree	161 (78.5%)
Annual household income	
£0–£20 000	12 (5.9%)
£21 000–£30 000	11 (5.4%)
£31 000–£40 000	14 (6.8%)
£41 000–£50 000	15 (7.3%)
£51 000–£60 000	18 (8.8%)
£61 000–£70 000	24 (11.7%)
£71 000+	111 (54.2%)
Financial situation during lockdown	
Living comfortably on income	103 (50.2%)
Coping on income	77 (37.6%)
Difficult on income	14 (6.8%)
Very difficult on income	9 (4.4%)
Not disclosed	2 (1.0%)
COVID-19 infection status of child	
Diagnosed and recovered	67 (32.7%)
Suspected and recovered	70 (34.1%)
No infection	68 (33.2%)
Suspected or diagnosed and still ill	0

The survey respondents comprised 189 mothers, 14 fathers and 2 other caregiving roles.

Means (SD) (ranges) and numbers (percentages) as relevant provided for each variable.

* 14 children in the sample were born between 32 and 36 weeks.

† n=193.

‡ The English Indices of Deprivation 2019 does not provide corresponding decile information for four participant postcodes. Data are only available for children assessed remotely.

§ n=1 participant had more than two parents/caregivers.

¶ Post-16 national qualifications in England.

** Post-18 national qualifications in England.

†† Children from London were over recruited as the majority of in-person testing took place in London schools; n=29 (16.48%) of the remote sample lived in London, which is in line with national data.

### Measures

Children were assessed at 4 years using five standardised assessments and two parent/caregiver questionnaires (from which we derived three measures) as part of a wider battery. Only measures with published norms available are reported here.

#### Direct assessments

Five areas were directly assessed by researchers using web-based digital versions of well-established clinical measures. The manualised instructions for administering and scoring these assessments were strictly followed (see online supplemental file 1 for information about normative datasets). Additional protocols were used to guide appropriate parental involvement. CDR (developmental psychologist) and JF (speech and language therapist) completed all assessments, and both were fully trained by LAH (clinical psychologist) and experienced with developmental measures.

Language was measured with the three Core Language Scales of the Clinical Evaluation of Language Fundamentals Preschool-second UK edition (CELF-Preschool-2-UK)^37^: Sentence Structure (understanding increasingly complex sentences), Word Structure (using language rules such as word endings or tense agreement) and Expressive Vocabulary (naming objects and actions). The British Picture Vocabulary Scale, third edition (BPVS-3)^38^ assessed receptive vocabulary (selecting the picture matching a spoken word). Non-verbal reasoning ability (recognising similarities and relationships between pictures and symbols) was assessed with the Matrices subtest of the British Ability Scales-3 (BAS-3).^39^

#### Caregiver-reported measures

Parents/caregivers completed questionnaire scales about their children’s executive functioning and motor skills (from which we derived three measures). The Behaviour Rating Inventory of Executive Function-Preschool (BRIEF-P)^40^ assessed overall EF (Global Executive Composite (GEC): inhibition, shifting, emotional control, working memory, planning and organising) and independent problem-solving (Emergent Metacognition Index (EMI): working memory, planning and organising). Motor skills (fine and gross—in daily life) were assessed using the Motor Scale of the Vineland Adaptive Behaviour Scales, third edition.^41^

### Procedure

Data collection took place from 1 May 2024 to 7 June 2025. In a planned comparison (see https://osf.io/27qhs),^42^ 25 children were assessed in person and 180 children remotely at home (figure 1) using identical protocols. No differences between remote and in-person assessment scores were found (see online supplemental file materials).

Children completed 3–4 assessment sessions lasting 45–60 min each for the wider test battery (occasionally more sessions were needed, max=10). Remote web-based assessments were completed via Zoom using an Amazon Fire 7 tablet (2022 release), which we provided, and families kept at the end of the study. In-person assessments were completed at school, and families received a voucher. The researchers administered the task battery in the same manner for both administration types, using a predetermined order, with breaks when needed. Parents/caregivers completed questionnaires covering demographics, home life during lockdown and their child’s current behaviour and development.

### Statistical analysis

On post hoc calculation, our sample (n=189 to n=205) was sufficient to detect small effect sizes (d*=*0.25) with 0.954 power using two-tailed α=0.05 for one sample and paired t-tests (G*Power).^43^ (See https://osf.io/27qhs^42^ for a priori calculations, which indicated n=200 for the more complex analyses planned for later phases of the study.)

Raw scores were transformed into age-related standardised scores using relevant manuals: T-scores (mean=50, SD=10) for Matrices and EF (GEC and EMI); standardised scores (mean=100, SD=15) for BPVS-3 and Motor Skills and scaled scores (mean=10, SD=3) for Sentence Structure, Word Structure and Expressive Vocabulary. To compare across measures, standardised scores were transformed into *z*-scores against published norms for each measure. For EF, *z*-scores were inverted because higher scores indicated less favourable outcomes.

The *z*-score distributions were assessed for skewness and kurtosis. All variables fell well within the Sattora-Bentler normality thresholds of ±2 on skewness and ±7 on kurtosis^44^ (recommended for samples n=200–500).^45^

One-sample t-tests explored differences between sample scores and published prepandemic norms. Percentages of children with lower scores are also reported. To benchmark skills in relation to non-verbal reasoning ability, paired t-tests compared each measure with matrices. Within research and practice involving children with developmental disorders, it is common to compare specific skills against a more global measure of general cognitive development, such as non-verbal reasoning, to identify and prioritise areas of need.

The number of datapoints varied for each measure (n=189 to n=205). Effect sizes and 95% CIs are reported. Significance was initially set at p<0.05, but Bonferroni correction for each research question (RQ) created an adjusted threshold of p<0.007. Note that this adjustment does not affect the pattern of significance. The wider study was preregistered (https://osf.io/27qhs),^42^ however, the current paper reports preliminary exploratory findings not listed in that document.

## Results

### RQ1: findings against prepandemic norms

One-sample t-tests demonstrated that (1) Sentence Structure, Expressive Vocabulary, BPVS-3 and Matrices were significantly above normative expectations for age; (2) Word Structure and Motor Skills were not different from normative expectations and (3) caregiver-reported EF (GEC and EMI) was significantly less favourable than normative expectations (see table 2).

**Table 2:** Descriptive statistics and one-sample t-test results for all study measures

Measure	N	Mean (SD) standardised scores	Mean (SD) *z*-scores	Expected population standardised score Mean (SD)	T	Df	P value	Cohen’s *d* (95% CIs)
Matrices								
(Non-verbal reasoning)	200	54.91 (11.62)	0.49 (1.16)	50 (10)	5.97	199	<0.001	d=0.42 (0.28 to 0 .57)
Sentence Structure								
(Receptive language)	205	11.69 (2.89)	0.56 (0.96)	10 (3)	8.37	204	<0.001	d=0.59 (0.44 to 0.73)
Word Structure								
(Expressive language)	204	10.02 (2.74)	0.01 (0.91)	10 (3)	0.10	203	0.919	d=0.01 (−0.13 to 0.14)
Expressive Vocabulary								
(Expressive vocabulary)	204	10.56 (2.33)	0.19 (0.78)	10 (3)	3.43	203	<0.001	*d*=0.24 (0.10 to 0.38)
British Picture Vocabulary Scale, third edition								
(Receptive vocabulary)	204	106.77 (13.87)	.45 (.93)	100 (15)	6.97	203	<0.001	d=0.49 (0.34 to 0.63)
Global Executive Composite*								
(Overall executive function)	190	55.91 (12.57)	−0.59 (1.26)	50 (10)	−6.48	189	<0.001	d=−0.47 (−0.62 to −0.32)
Emergent Metacognition Index								
(Independent problem-solving)	190	54.43 (12.16)	−0.44 (1.22)	50 (10)	−5.02	189	<0.001	d=−0.36 (−0.51 to −0.22)
Vineland Motor Scale								
(Motor skills)	189	100.97 (10.94)	0.06 (0.73)	100 (15)	1.22	188	0.223	d=0.09 (−0.05 to 0.23)

Mean (SD) for standardised scores and *z*-scores are provided.

* Higher scores indicate a less favourable outcome.

Table 3 shows the number of individual children with below-average scores (falling between –1 SD and –2 SD; expected rate=14%) and very low scores (at or below –2 SD; expected rate=2%) from the prepandemic normative mean. For word structure and EF (GEC and EMI), blue shading indicates higher than expected numbers of children scoring at lower levels.

**Table 3:** Number (percentage) of children falling between –1 SD and –2 SD from published norm means and at or below −2 SD from published norm means

Measure	N	Number (%) of children between –1 SD and −2 SD	Number (%) of children at or below −2 SD
Matrices			
(Non-verbal reasoning)	200	16 (8.0%)	0 (0.0%)
Sentence Structure			
(Receptive language)	205	10 (4.9%)	5 (2.4%)
Word Structure			
(Expressive language)	204	18 (8.8%)	11 (5.4%)
Expressive vocabulary			
(Expressive Vocabulary)	204	16 (7.8%)	4 (2.0%)
British Picture Vocabulary Scale, third edition			
(Receptive vocabulary)	204	9 (4.6%)	4 (2.0%)
Global Executive Composite			
(Overall executive function)	190	34 (18.0%)	29 (15.2%)
Emergent Metacognition Index			
(Independent problem-solving)	190	43 (22.6%)	20 (10.6%)
Vineland Motor Scale			
(Motor skills)	189	13 (6.9%)	0 (0.0%)

Blue shading indicates higher than expected numbers of children observed falling into each category (residual >7; other cell residuals were all <2). For χ2 analyses, residuals of two and above represent cells where the observed count is significantly higher than the expected count. Although they are not effect sizes per se, they help to unpick the overall result and identify which cells are driving the result.

### RQ2: findings using non-verbal reasoning ability as a benchmark

We then benchmarked areas of development against domain-general non-verbal reasoning to assess whether particular skills showed strengths and weaknesses compared with cognitive potential. Sentence Structure and BPVS-3 were not significantly different from Matrices (Sentence Structure: t(199)=1.09, p=0.276, d=0.08 (−0.06 to 0.22); BPVS-3: t(199)=-0.17, p=0.868, d=−0.01 (−0.15 to 0.13)). All other measures were significantly below Matrices (Word Structure: t(198)=−4.94, p<0.001, d=−0.35 (−0.49 to −0.21); Expressive Vocabulary: t(198)=−3.13, p=0.002, d=−0.22 (−0.36 to −0.08); EF: (GEC, t(188)=−9.86, p<0.001, d=−0.72 (−0.88 to −0.56); EMI, t(188)=−8.64, p<0.001, d=−0.63 (−0.78 to −0.47)) and Motor Skills: t(187)=−4.54, p<0.001, d=−0.33 (−0.48 to −0.18)). See figure 2.

**Figure 2:**
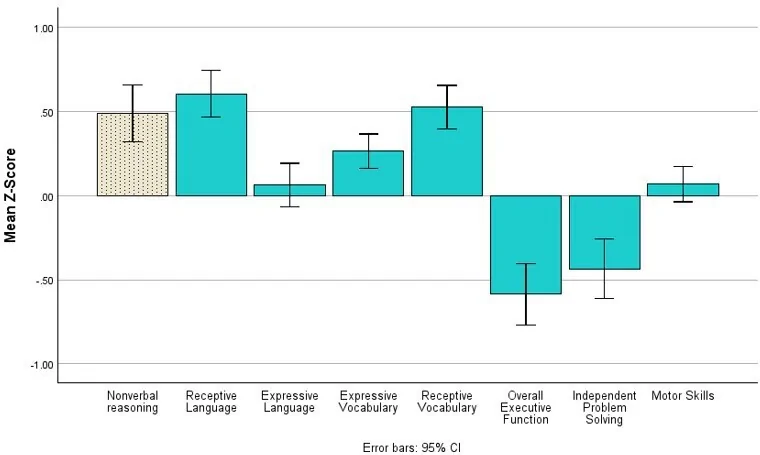
Comparisons between z-scores for all variables, with 95% CIs. Note: All variables are shown in relation to expected level for age (represented by z=0) according to their individual test norms (see online supplemental file 1). Non-verbal reasoning is shaded differently from all other variables as this was used as a comparative benchmark for research question 2.

## Discussion

This is the first study to examine language, EF, motor skills and non-verbal reasoning in a large sample of 4-year-old children born during the first COVID-19 lockdown in England. These preliminary findings indicate that both indices of caregiver-reported EF (GEC and EMI) were significantly below normative expectations, although receptive language (Sentence Structure and BPVS-3), Expressive Vocabulary and non-verbal reasoning ability (Matrices) exceeded prepandemic normative levels. Word Structure and Motor Skills were in line with normative expectations. At the individual level, more children than expected showed significant EF needs; more also obtained very low scores on Word Structure, although other areas of language were as expected. We also benchmarked specific areas of development against a more domain-general measure of non-verbal ability, an approach often used to identify primary needs when addressing developmental conditions. Using this approach, EF was again below cognitive potential and, additionally, language results showed more nuanced patterns: receptive language (Sentence Structure and BPVS-3) was comparable to the benchmark, whereas expressive language (Word Structure and Expressive Vocabulary) fell significantly below. Motor skills also fell below non-verbal reasoning.

The most noteworthy findings concerned caregiver-reported EF. Whether compared with prepandemic norms or the benchmark of non-verbal reasoning, overall EF (GEC) and independent problem-solving (EMI) were lower than expected. Furthermore, at the individual level, one-third of children in this born-in-lockdown sample were rated as having EF needs. These findings echo recent research using the BRIEF-P caregiver questionnaire with pandemic and prepandemic samples.^30^ Other studies have also reported pandemic impacts on EF in young children born before or during the wider pandemic timeframe.^16–22^ For example, toddlers who attended high-quality early childhood education in 2020, just as they experienced abrupt and extended disruptions, had lower EF compared with toddlers in prepandemic and postpandemic attendance groups.^18^ Slower pandemic-related growth of EF has also been noted in several studies.^16 21 22^

However, the present study is the first, to our knowledge, to include a large sample of school-age children born in lockdown in England. The findings suggest that limited social diversity in the early years has impacted everyday EF.^9^ Children may find goal-directed, planful behaviour more difficult, for example, settling and keeping on task, remembering/following instructions, resisting distraction or changing behaviour based on feedback. These needs may lead to ‘vulnerable’ classrooms, which require responsive educational policy and targeted support strategies.

A more uneven picture was evident for language. Overall, language scores were at or above expected levels for age. Interestingly, when comparing the language measures with a benchmark of non-verbal reasoning ability, a clear split emerged between receptive and expressive language skills. Receptive language was on a par with non-verbal reasoning, whereas expressive language was below it; and more individual children than expected scored below –2 SD from the mean on Word Structure (5.4% compared with the expected 2%). Word Structure requires the ability to use word rules, use word endings, create new words from base words and use pronouns to talk about oneself or other people. The current preliminary findings suggest that expressive language is somewhat more vulnerable to pandemic impacts than receptive language, possibly due to the loss of rich and varied interactions with less familiar people, which in turn may impact expressive semantic and syntactic development.^10^

Given the importance of early social environment for communication, the language results from the present study are surprising, as they evidence fewer pandemic-related language difficulties than some previous work on infants and toddlers^12 14 27 29 31–34 46^ (although see Kartushina *et al*^15^ and Sade *et al*^30^). This may be because language is highly robust and could have largely caught up by the age of 4 years, despite environmental changes. It could also be that receptive language has been relatively well supported by increased within-family interaction during the first year,^17^ whereas more diverse exposure to optimise expressive language development^10^ was restricted. Interestingly, Sperber *et al* (2022) reported that language development was not significantly related to length of lockdown exposure in the first 18 months,^2^ so the causal pathways are likely to be complex. For example, language needs may only become evident in classroom settings once social interaction demands are increased.

The relatively weaker expressive language and lower EF could be the result of more limited social opportunities experienced in the first year. For example, learning to communicate and manage behaviour in public spaces was absent during lockdown periods. In contrast, receptive language may have been somewhat protected by increased familial attention and communication. These possible associations need further research.

Turning to parent-reported motor skills, the present born in lockdown group met prepandemic normative expectations for age, and no more children than expected fell below 1 SD from the prepandemic mean. However, motor skills were significantly below the non-verbal reasoning benchmark. Nevertheless, the picture from previous research is complex: children born in lockdown show lower motor skills at 20 months,^36^ yet there have been reports of improvements post-pandemic in fine motor skills for children born before lockdowns.^26^ Furthermore, younger children in a mixed-age sample born before the pandemic fared better on age-appropriate motor measures than children who had been older at the start of lockdown.^24^ Given the importance of motor development to the development of language, social and other cognitive skills, further research is needed.

### Strengths and limitations

The present study is the most comprehensive to date to report on language, EF, motor skills and non-verbal reasoning in a large sample of 4-year-old children born during the first lockdown in England. Our assessments were thorough and used widely respected, in-depth measures. Remote testing helped to remove barriers to participation and widen inclusiveness: we were successful in recruiting families from all deciles of multiple deprivation and all regions of England. We used the same device and the same two researchers for all children to limit variability. The sample size and tight birth-date parameters were also strengths.

However, participating families had higher educational levels than the general English population (~48% holding a degree in this parental age bracket), which could have led to some of the above-average scores. Another factor supporting children’s performance may be remote testing. To mitigate this, strict protocols were used to prevent caregiver help during remote assessments, and our analyses showed no differences between remote and in-person administration types (online supplemental file 1; see also Nelson *et al*^47^ who found no evidence of parent-support effects).^47^ Furthermore, remote testing has been shown to be valid and accurate.^32 48^ Relatedly, we accept that using prepandemic norms rather than a matched comparison group is a central limitation to this preliminary analysis, since the demographics of children represented in those norms may be different from our own sample. Another factor to note is that BRIEF-P (and Vineland Motor Skills) norms were based on US children, and while we have no reason to expect large cross-national differences, this also should be considered when interpreting the findings. Future research with similar samples will help to disentangle demographic effects, as well as alternative explanations such as parental stress, screen use and more widespread postpandemic shifts in child behaviour.

Importantly, EF and motor skills were measured via caregiver report and were not directly assessed. In later phases of this study, direct measures of executive functioning will be available for comparison with children born after lockdown (https://osf.io/27qhs).^42^ We acknowledge that results could be biased by parent factors such as their own mental health or overall experiences of parenting. However, the BRIEF-P is a very well-established standardised tool, widely used in research. Furthermore, caregivers were not similarly negative on the motor scale, which was also caregiver-reported and scored using US norms.

In our self-selected sample, it is plausible that participating caregivers were those most concerned about their children’s progress. However, the sample was stratified for SES and geographic region; families were rewarded with a free tablet for their time (encouraging parents of a wide range of children to sign up); and only the BRIEF-P scores show marked differences from expectations. We found most scores met or exceeded expected levels for age; thus, the stand-out scores from the BRIEF-P are still of interest. Furthermore, the BICYCLE study is completing qualitative interviews with a subset of caregivers to triangulate the quantitative findings.^49^

Finally, the wider BICYCLE study was designed to compare scores of children born in lockdown with children born after lockdown. Other factors may have affected developmental pathways, including demographic characteristics and more permanent societal changes. The additional planned postlockdown sample will further enrich our understanding.

## Conclusion and implications

Caregiver-reported EF was the most noteworthy area of concern for children born in lockdown. Expressive language and motor skills were also impacted in relation to cognitive potential. The present study is the first to report on a large-scale, directly tested sample of 4-year-old children who were born *during* lockdowns (rather than before) and to use a novel benchmarking approach. Our sample also represented a diverse range of demographic, ethnic and geographic backgrounds across England.

Unlike some studies of children before and during the wider pandemic timeframe and results from those born in lockdown at earlier ages, we found that the language and motor skills of 4-year-olds born in lockdown exceeded or met expected levels. However, our results are in line with emerging evidence for EF support needs in children born before or during the pandemic.

Children who were born in lockdown may find themselves in classrooms where a significant number of individuals are dysregulated and who struggle to express themselves as effectively as their cognitive abilities would suggest. The present results imply the need for additional classroom scaffolding for behavioural regulation, EF-focused approaches and more explicit strategies for expressive language, rather than individual screening or specialist support.

Our findings may also inform support for other young children already born when lockdowns began, those outside of the UK who experienced similar restrictions and the next wave of children born after lockdowns had ended. However, further research is needed to investigate optimum ways to support this generation of children. The preliminary BICYCLE findings represent an important first step in understanding and supporting the effects of extreme social restrictions in the first year of life.

## Supplementary material

10.1136/archdischild-2025-329529 online supplemental file 1

## Data Availability

No data are available.
