# Supplementary material for: Developmental outcomes in 4-year-old children born in the first COVID-19 lockdown in England: preliminary findings from the Born In COVID Year – Core Lockdown Effects (BICYCLE) study
Source: Arch Dis Child. 2026 Jul 14;111(10):e329529. doi: 10.1136/archdischild-2025-329529 (PMC13638306; doi:10.1136/archdischild-2025-329529)
Supplement: Supplementary file 1 [file archdischild-111-10-s001.docx]

**Supplementary Materials for:**

“Developmental outcomes at four-years-old in children born in the first Covid-19 lockdown in England: Preliminary findings from the Born In Covid Year – Core Lockdown Effects (BICYCLE) study”

^1^Botting, N., ^1^Del Rosario, C., ^1^Flynn, J., ^1^Reimers, S., ^2^Davies, C., ^3^Gonzalez-Gomez, N., ^4^Hendry, A., ^1^Hill, E.L., ^5^McGillion, M., ^1^Moss, B., ^6^Shapiro, L. & ^1^Henry, L.A.

**Affiliations:** ^1^City St George’s, University of London; ^2^University of Leeds; ^3^Oxford-Brookes University; ^4^University of Oxford; ^5^University of Warwick; ^6^Aston University

**Purpose of Supplementary Materials**:

These Supplementary Materials report on: (1) analyses investigating whether administration type (remote or in-person) had an effect on language, executive function, motor skills, or non-verbal reasoning in children born-in-lockdown who were tested as part of the BICYCLE study; and (2) further information about the normative samples for standardised tests used in this study.

(1) Comparing children tested remotely and in-person

A total of 205 four-year-old children from across England were included in the born-in-lockdown sample of the BICYCLE study. Most of these (n=180) were tested remotely via Zoom, but the remainder were tested in their schools in-person (n=25). A comparison of remote versus in-person administration types was planned and pre-registered (see [https://osf.io/27qhs)](https://osf.io/27qhs)^43^.

To determine whether in-person and remote testing were equivalent in terms of the scores on assessments of nonverbal reasoning, language, executive function and motor skills, all children tested in the same region of England that provided largest amount of relevant data (London region: in-person n=21; remote n=29) were included in the analyses reported below. These sub-sample sizes give an acceptable to good post-hoc power of 0.89 for detecting any two-tailed large effects of administration type in our regression analyses (*f*^2^>0.35) with α =0.05 using G*Power3.1^44^. We are therefore confident that any substantial differences caused by administration type will be detected. However, we accept that more subtle differences may not be captured. Note that measures of nonverbal reasoning and language were directly administered tests, whereas measures of executive function and motor skills were caregiver-report questionnaires. It is arguable that differences between remote and in-person groups would not be expected on the caregiver report questionnaires because all caregivers completed the questionnaires in their own time outside the child assessment sessions. Nevertheless, we include these measures for completeness given there were minor differences in terms of how families were recruited in the remote versus in-person groups.

*Comparing remote and in-person assessment groups on demographic variables*

Demographic variables (child age at assessment, child gestational age, number of siblings, annual household income, number of people in household, child gender, child ethnicity, child multiple languages, number of people with parental responsibility, and maternal education) were investigated to determine whether the in-person and remote groups were comparable. Group comparisons were carried out using *t*-tests, Mann-Whitney *U* tests, or Chi-Squared tests for scale, ordinal, and categorical data respectively. Given multiple tests, a more conservative significance value (*p* < .01) was used.

For child age at assessment and gestational age, *t*-tests showed no significant administration type group differences (child age at assessment, *t*(48) = -1.97, *p* = .054; child gestational age, *t*(48) = 1.90, *p* = .063). Mann-Whitney *U* tests showed significant assessment type group differences for number of siblings (*U* = 456.5, *z* = 3.35, *p* < .001) and annual household income (*U* = 153.5, *z* = -3.67, *p* < .001), but not for number of people in household (*U* = 412.5, *z* = 2.38, *p* = .017). In-person participants had families with larger numbers of siblings and lower annual household income.

Chi-Squared tests indicated no significant assessment type group differences for the remaining demographic variables (child gender, χ*^2^* (1) = 0.27, *p* = .603; child ethnicity, χ*^2^* (4) = 13.09, *p* = .011; multiple languages (child), χ *^2^* (1) = 5.63, *p* = .018; number of people with parental responsibility, χ *^2^* (1) = 1.41, *p* = .235; or maternal education, χ *^2^* (2) = 8.27, *p* = .016).

*Comparing remote and in-person groups on assessment measures*

To investigate whether administration type had any effect on the assessment measures of non-verbal reasoning, language, executive function or motor skills, hierarchical linear regression analyses were carried out with each of these measures as a dependent variable. The regression analyses included the demographic variables that differed between the remote and in-person groups as control variables at step 1 (number of siblings, annual household income, child ethnicity), and administration type (remote vs in-person coded as a dummy variable) as a predictor at step 2. Note that as the group difference in child ethnicity was on the borderline of significance, this variable was also included as a control variable in the regressions.

One regression for each of the eight measures of interest was run (non-verbal reasoning, Sentence Structure, Word Structure, Expressive Vocabulary, BPVS-3 Receptive Vocabulary, Global Executive Composite, Emergent Metacognition Index, Vineland Motor Scale). Table S1 summarises information from step 2 of all regressions.

Of key importance, there was no significant effect of administration type at step 2 for any measure, with at most 6% (range .5% to 6%) of the total variance in assessment score being accounted for when this predictor was included. Therefore, there was no evidence that administration type had any effect on children’s scores after controlling for demographic differences between the groups.

Furthermore, the overall regression models were significant for only three measures (Sentence Structure, *F* (4,45) = 3.31, *p* = .019; Expressive Vocabulary, *F* (4,45) = 3.14, *p* = .023; and BPVS-3, *F* (4,45) = 3.20, *p* = .021). Examining the beta coefficients revealed that annual household income was a significant predictor (only at step 1) in two regressions (Sentence Structure, *p* = .024; Word Structure, *p* = .040) but that this variable did not remain significant at step 2. No other control variables reached significance in any step in any regression.

Table S1: Summary information for regressions looking at the effect of administration type (remote versus in-person) on non-verbal reasoning, language, executive function and motor skills in children from the born-in-lockdown sample of the BICYCLE study in the London region.

| Measure | Total unadjusted R^2^ accounted for by the full model | Change in R^2^ at step 2 | β Ethnicity | β Siblings | β Income | β remote vs. in-person |
| --- | --- | --- | --- | --- | --- | --- |
| Matrices | .15 | .06 | -.13 | -.003 | .36 | .28 |
| Sentence Structure | **.23*** | .03 | -.11 | .12 | .34 | -.21 |
| Word Structure | .17 | .005 | -.17 | .21 | .35 | -.09 |
| Expressive Vocabulary | **.22*** | .04 | -.14 | .12 | .27 | -.24 |
| BPVS-3 | **.22*** | .005 | -.24 | -.13 | .23 | .08 |
| Global Executive Composite | .09 | .03 | .14 | -.11 | -.30 | -.20 |
| Emergent Metacognition Index | .09 | .01 | .16 | -.16 | -.30 | -.13 |
| Vineland Motor Scale | .12 | .05 | -.11 | -.09 | .28 | .26 |

**p*<.05

(2) Further information about the normative samples for standardised tests used in this study

The standardised measures used in this study are supported by robust normative data with strong psychometric properties. The CELF-Preschool 2 was used to assess three areas of language. This test was validated in the UK using a sample of 486 children and demonstrates excellent test–retest reliability (0.91–0.94^)38^. The BPVS-3, used to assess receptive vocabulary, was standardised on a large UK sample of 3,278 children, and shows excellent internal reliability (0.91)^39^. Non-verbal ability was assessed using the Matrices subscale of the BAS-3, which was standardised on 1,480 UK children. The Matrices subscale demonstrates strong internal consistency (0.84)^40^. These three UK normative samples were stratified by region, sex, ethnicity, and parental education, with the CELF-Preschool 2 and BAS-3 in line with the 2001 UK Census.

Both the BRIEF-P (used to assess two indices of executive function) and the Vineland-3 (used to assess motor skills) are based on US normative samples. The BRIEF-P was normed on 460 parents of children aged 2 to 5 years in the United States. It demonstrates acceptable to excellent reliability, with test–retest correlations ranging from 0.78 to 0.90^41^. The Vineland-3 Motor Scale was validated using a normative sample of 2,560 children and shows excellent internal consistency (0.90)^42^. Both normative samples were representative of the US population taking into consideration gender, socioeconomic status, ethnicity, and geographic location.
